# Familiarity and Perceptions of Aducanumab in Caregivers of Hawaii Alzheimer’s Disease Patients: Results of a Telephone Survey

**DOI:** 10.7759/cureus.50001

**Published:** 2023-12-05

**Authors:** Connor Goo, Frances Morden, Kasen Wong, Sarvia Aquino, Jaron Kawamura, Vanessa Rubel, Samantha Masca, Rachel Gorenflo, Enrique Carrazana, Kore Liow

**Affiliations:** 1 Brain Research, Innovation & Translation Laboratory, Hawaii Pacific Neuroscience, Honolulu, USA; 2 Neurology, John A. Burns School of Medicine, University of Hawaii at Manoa, Honolulu, USA

**Keywords:** phone survey, caregiver, hawaii, aduhelm, aducanumab, alzheimer's disease

## Abstract

Aim: To identify current perceptions of aducanumab among patients with Alzheimer’s disease (AD) and their caregivers.

Methods: A total of 352 caregivers of AD patients seen at Hawaii’s largest multidisciplinary neuroscience center between January 01, 2019, and June 21, 2021, were surveyed by telephone to understand patient and caregiver knowledge, familiarity, and hesitancy toward aducanumab.

Results: Thirty-seven percent of caregivers were familiar with aducanumab. Caregivers who were spouses of their respective patients with AD (p=0.0023) had increased odds of familiarity. Additional predictors of aducanumab familiarity included patients with higher mini-mental state examination scores (p=0.0076) and those who received mental stimulation (p=0.007). Conversely, caregivers who identified as native Hawaiian and other Pacific Islanders (NHPI) (p=0.044) or the patient’s child (p=0.010) were predictors of decreased familiarity. Only 33% of caregivers familiar with aducanumab believed it to be safe and 56% reported “side effects” as their top concern. Thirty percent of caregivers were moderately ready or very ready to use aducanumab if given the opportunity.

Conclusion: Most caregivers of Hawaii AD patients were unfamiliar with aducanumab. Furthermore, those familiar were hesitant to trial the medication. Improved education and awareness of AD therapies are important, so families and caregivers of AD patients can make more informed decisions regarding AD treatment.

## Introduction

Aducanumab (Aduhelm), a monoclonal antibody treatment from Biogen®, received accelerated approval by the Food and Drug Administration (FDA) on June 7, 2021, due to evidence of it removing beta-amyloid deposits in the brain, a surrogate biomarker for AD progression [1]. Results showed a dose-and-time-dependent reduction in beta-amyloid pathology with aducanumab in phase 3 studies of EMERGE but not ENGAGE [2]. The FDA's approval of aducanumab caused reluctance and criticism with inconclusive results for demonstrating improvement in clinical endpoints [3]. Due to the approval of this treatment, acknowledging the public’s perception is necessary to better inform successful drug implementation strategies and address concerns expressed by patients with AD and their caregivers [4]. There exists a considerable information gap with the accelerated FDA approval process, uncertainty surrounding clinical benefits, and patient information and recommendations by physicians grappling with the controversy and progress of aducanumab [5]. Previous surveys measuring knowledge and attitudes toward aducanumab overrepresent non-Hispanic White individuals and conclude a lack of broad understanding or enthusiasm associated with the drug’s publicity alone [6]. Shortly after aducanumab’s approval and during its peak publicity, caregivers of patients with AD at Hawaii’s largest multidisciplinary neuroscience institution were surveyed to gauge knowledge and perception of the drug with the goal of determining patient subsets who may require further education and resources.

## Materials and methods

This quality improvement (QI) study utilized a telephone survey to understand patient and caregiver knowledge, familiarity, and hesitancy toward aducanumab. The QI survey was conducted between July 17, 2021, and July 31, 2021, with caregivers of all patients with AD who were seen at Hawaii Pacific Neuroscience (HPN). While HPN receives research funding from Biogen®, there was no financial incentive for survey participation. All participants provided verbal informed consent after disclosure of survey risks, benefits, objectives, and anonymity prior to survey administration. Patient medical records were reviewed, and all data were de-identified.

Survey design

The survey was developed following consultation with a multidisciplinary team of clinicians, including geriatricians and neurologists. All calls were in English and followed a structured script of up to 27 questions. The 10-minute survey included questions regarding AD patients’ current management, overall disease progression, and caregivers’ familiarity with aducanumab (Table 1). Caregivers familiar with the drug were then asked questions specific to aducanumab, including their perception of medication safety, concerns, and readiness to incorporate aducanumab into their respective patient’s treatment plans (Table 2). All caregivers were also asked to identify their patient’s primary medical decision-maker and provide demographic information for the patient and caregiver.

**Table 1:** Caregiver and AD patient sociodemographics and patient care characteristics.

	Number of caregivers familiar with aducanumab	Number of caregivers unfamiliar with aducanumab
Caregiver race		
NHPI	3	18
White	9	9
Asian	15	24
Hispanic	0	1
Caregiver status		
Self	3	5
Spouse	16	9
Child	9	27
Sibling	1	3
Other Family	1	3
Caregiver	1	7
Caregiver surveyed		
Primary caregiver	28	49
Not primary caregiver	1	3
Caregiver education		
High school/GED	3	8
Some college	3	6
Associate/Bachelors	14	34
Master’s	7	4
Doctorate	2	0
Caregiver typical source of health information		
Social media	0	0
Media news (television, radio, articles)	9	15
Friends/family/coworkers	2	3
Healthcare provider	10	27
Scholarly articles/government websites	8	6
Caregiver heard recent news about Alzheimer's		
Heard recent news about Alzheimer's	28	20
Did not hear recent news about Alzheimer's	2	34
Patient sex		
Male	14	18
Female	17	36
Patient race		
NHPI	2	12
White	11	14
Asian	14	24
Hispanic	0	2
Patient zip code income quartile		
Quartile 1	6	3
Quartile 2	6	17
Quartile 3	7	14
Quartile 4	12	20
Patient insurance type		
Medicare	26	49
Medicaid	3	4
Private	1	1
Patient marital status		
Single	3	4
Married	14	22
Divorced	0	4
Widowed	7	14
Difficulty of patient care (1 being not difficult at all, 5 being most difficult)		
1	4	5
2	6	5
3	10	16
4	6	10
5	1	13
How has Mr./Ms. _____'s current treatment affected their condition?		
Better	7	12
Same	14	25
Worse	3	11
Cholinesterase inhibitor		
Cholinesterase inhibitor	16	25
No cholinesterase inhibitor	15	29
Glutamate regulator		
Glutamate regulator	11	24
No glutamate regulator	20	30
Over the counter supplements		
OTC supplements	16	14
No OTC supplements	14	33
Lifestyle change: healthy diet changes		
Healthy diet changes	25	41
No healthy diet changes	4	11
Lifestyle change: regular exercise		
Regular exercise	20	27
No regular exercise	9	25
Lifestyle change: mental stimulation		
Mental stimulation	25	29
No mental stimulation	4	23
Lifestyle change: social engagement		
Social engagement	21	35
No social engagement	8	17
Lifestyle change: quality sleep		
Quality sleep	24	33
No quality sleep	5	19
Lifestyle change: stress management		
Stress management	20	22
No stress management	8	29

**Table 2:** Aducanumab-specific survey questions administered to caregivers of AD patients indicating familiarity with the monoclonal antibody.

Aducanumab specific survey questions and number of responses (percentage)	
Do you (caregiver) feel well-informed about Aduhelm (aducanumab)?	
Yes	17 (54.8%)
No	14 (45.2%)
Do you (caregiver) feel well-informed about Aduhelm's (aducanumab's) side effects?	
Yes	19 (61.3%)
No	12 (38.7%)
Do you (caregiver) believe Aduhelm (aducanumab) is safe?	
Yes	5 (33.3%)
No	10 (66.7%)
Are you (caregiver) reluctant or hesitant about Mr./Ms. _____ receiving Aduhelm (aducanumab)?	
Yes	22 (81.5%)
No	5 (18.5%)
Among this list of factors, please confirm whether each factor regarding Aduhelm (aducanumab) concerns you (caregiver) by responding with yes or no:	
Safety regarding intravenous administration	
Yes	10 (40.0%)
No	15 (60.0%
Safety regarding side effects	
Yes	21 (77.8%)
No	6 (22.2%)
Expedition of approval	
Yes	16 (64.0%)
No	9 (36.0%)
Interference with current treatment	
Yes	11 (40.7%)
No	16 (59.3%)
Patient's current health status	
Yes	14 (58.3%)
No	10 (41.7%)
Accessibility	
Yes	10 (43.5%)
No	13 (56.5%)
Finances	
Yes	18 (69.2%)
No	8 (30.8%)
Which of the following would you (caregiver) say is your top concern?	
Safety regarding intravenous administration	2 (8.0%)
Safety regarding side effects	14 (56.0%)
Expedition of Aduhelm's (aducanumab's) approval	2 (8.0%)
Interference with current treatment	2 (8.0%)P
Patient's current health status	1 (4.0%)
Accessibility	0 (0.0%)
Finances	4 (16.0%)
If finances were not an issue, would you (caregiver) have Mr./Ms. _____ take Aduhelm (aducanumab)?	
Yes	10 (43.5%)
No	13 (56.5%)
Would you (caregiver) be willing to have Mr./Ms. _____ undergo additional testing for Alzheimer’s in order to qualify for insurance coverage for this treatment?	
Yes	15 (65.2%)
No	8 (34.8%)
Do you (caregiver) believe Mr./Ms. _____’s current treatment regimen is more effective than Aduhelm (aducanumab)?	
Yes	4 (36.4%)
No	7 (63.6%)
Would you (caregiver) be willing to have Mr./Ms. _____ participate in clinical trials for Aduhelm (aducanumab) or would you (caregiver) rather wait for the follow-up trials before having them receive the treatment?	
Participate	7 (33.3%)
Wait	14 (66.7%)
What would you (caregiver) say is your readiness to have Mr./Ms. _____ receive Aduhelm (aducanumab) if given the opportunity?	
Very ready	5 (16.7%)
Moderately ready	4 (13.3%)
Neutral	4 (13.3%)
Not ready to take	8 (26.7%)
Will not take	9 (30.0%)
Do you (caregiver) intend to have a discussion about the risks and benefits of receiving Aduhelm (aducanumab) with Mr./Ms. _____'s healthcare provider?	
Yes	16 (55.2%)
No	8 (27.6%)
Already discussed with the provider	5 (17.2%)

Study population and data collection

Participants included caregivers of all patients with AD who were seen at HPN between January 1, 2019, and June 22, 2021, excluding caregivers of patients with AD who were deceased at the time of the survey. Patients with AD who cared for themselves were considered their own caregivers. Sociodemographic data, including age, insurance type, race, sex, zone improvement plan (ZIP) code, and medical comorbidities, were also collected from each patient’s electronic medical records. The ZIP code served as a proxy measure for the median household income and population size of each patient’s municipality [7].

Statistical analysis

Bivariate analyses utilized nonparametric testing. Categorical variables were assessed with Pearson’s chi-squared test or Fisher’s exact test of independence for sample sizes less than five with Haldane-Anscombe correction, as necessary [8-12]. These were reported as odds ratios (OR) with 95% confidence intervals (95% CI). Each OR represented the odds of being familiar with aducanumab. Continuous variables were assessed by the independent Wilcoxon rank sum test. Multivariable logistic regression models with Firth’s correction were utilized to identify factors that independently predicted familiarity with aducanumab [13]. All tests were two-tailed with an alpha level of <0.05 being considered statistically significant. Analyses were conducted through R statistical software (R Foundation for Statistical Computing, Vienna, Austria) [14].

## Results

Survey respondents

Of the 352 patients identified with AD, 13 patients were deceased, and 339 were alive at the time of the survey. Eighty-six (25.4%) survey responses out of 339 eligible caregivers were collected. Fifty-four (62.8%) caregivers were unfamiliar with aducanumab, while 32 (37.2%) caregivers were familiar with the drug. Familiarization with aducanumab was found to be greater for caregivers who were spouses of their respective patients with AD (OR: 5.21, 95% CI: 1.75, 16.55; p=0.002), heard about AD recently in the news at the time of the survey (OR: 22.91, 95% CI: 4.93, 218.10; p<0.001), or received a master’s degree (OR: 3.75, 95% CI: 0.85, 19.36; p=0.049). Additionally, caregivers of patients with higher mini-mental state examination (MMSE) scores (difference of sums: 3.00, 95% CI: 1.00, 6.00; p=0.022), patients using stress management techniques (OR: 3.24, 95% CI: 1.12, 10.22; p=0.030), patients who receive mental stimulation (OR: 4.87, 95% CI: 1.40, 21.97; p=0.007), and patients who were former smokers (OR: 4.03, 95% CI: 1.35, 12.62; p=0.009) were more likely to be familiar with aducanumab. Of the 32 caregivers familiar with aducanumab, only 33% believed it to be safe, and 56% of caregivers chose safety regarding side effects as the top concerning factor of aducanumab. Only 30% of caregivers were moderately ready or very ready for their respective care recipient with AD to receive aducanumab.

Multivariable logistic regression

Multivariable analysis identified factors that predicted aducanumab familiarity. Caregivers of AD patients with higher MMSE scores (OR: 2.22; p=0.0076), patients receiving mental stimulation (OR: 699.11; p=0.033), and patients using OTC supplements (OR: 1.21x10^10^; p=0.010) had increased odds of aducanumab familiarity. Caregivers who were native Hawaiian and other Pacific Islanders (NHPI) (OR: 9.66x10^-5^; p=0.044) or were children (OR: 2.25x10^-6^; p=0.010) of the AD patient had decreased odds of being familiar with aducanumab. Caregivers with “other” (sibling, other family members, or nonrelative) relationships to the patient with AD also had lower odds of being familiar with aducanumab (OR: 6.14x10^-4^; p=0.041).

Survey non-respondents

Survey non-respondents and respondents were similar for patient age, sex, race, insurance type, population density, population size, median household income, and MMSE. Caregivers of AD patients from impoverished areas (overall poverty level in municipality, difference of sums: 4.00x10^-3^, 95% CI: 5.78x10^-5^, 7.08x10^3^; p=0.025) and poverty level for ages 65+ (difference of sums: 2.99x10^-3^, 95% CI: 1.30x10^-5^, 5.04x10^3^; p=0.043) were less likely to respond to the phone survey.

## Discussion

To the best of our knowledge, this study is among the first to survey caregivers of patients with AD on their familiarity and perceptions of aducanumab. Only 37.2% of caregivers who completed our survey were familiar with aducanumab, similar to a 2022 online survey, in which 25% of their respondents (adults nationwide aged 35 years and older) reported initial familiarity [15]. In contrast to prior surveys, this is one of the first studies to directly contact caregiver units of patients with AD, rather than the general population, so it is the first to characterize familiarity based on the caregiver’s relationship with the patient. Importantly, as evidenced by literature regarding the retention of AD patients in clinical trials, the caregiver plays a crucial role in the patient’s continued participation in treatment trials [16].

Our results demonstrated a decreased likelihood of aducanumab familiarity among NHPI caregivers and caregivers who were children of the AD patient. Compared to previous surveys regarding aducanumab in which the NHPI population comprised less than 3.4% of respondents [6,15], our survey included a robust number of NHPI respondents that encompassed 26.6% of all respondents. NHPI are severely underrepresented in medical studies and clinical trials, previously having their data aggregated with Asian Americans and thus misreporting their race-associated outcomes [17]. Previous studies have found perceived financial stress to be associated with cost-related treatment nonadherence behavior trends in NHPI populations [18]. Other studies note cultural safety, an intention to learn about diverse cultures, consideration of historical contexts of different ethnic groups, and emphasizing cautious communication with NHPI patients when introducing treatment regimens [19]. Insight into direct opinions concerning treatments will further illuminate both concerns and barriers to considering aducanumab.

The majority of caregivers who were familiar with aducanumab reported “safety regarding side effects” as the most concerning factor regarding the drug, whereas a smaller subset reported medication cost as their top concern. DiStefano et al. noted a general willingness (60%) to enroll a family member in a randomized placebo-controlled trial for aducanumab and a general unwillingness to pay certain premium increases [15]. These discrepancies may point to different survey populations, with our study’s caregivers being more directly involved in treatment finances than the age-stratified general public populations in online surveys.

Absolute generalization remains limited due to a small sample size compared to other survey studies on aducanumab. While such surveys were automated and incentivized, few investigated aducanumab hesitancy and knowledge through survey administration, none were conducted through phone interviews, and none directly contacted family units of AD patients. Since this study was conducted in Hawai’i, wherein the ethnocultural and racial population makeup is strikingly different to the rest of America, these results might be extrapolated to parts of the country with more ethnocultural and racial homogeneity [20]. Additionally, aducanumab is more widely used and available, with many patients enrolled in the Biogen Phase IV ENVISION trial. Sentiments may be changing, and our group aims to collect follow-up data for comparison. In addition, future studies may compare hesitancy and knowledge of developing medications with the current standard of care.

## Conclusions

Overall, our study suggests that patients and caregivers living with AD in Hawai’i have concerns about aducanumab’s efficacy, safety, and costs. Most caregivers are not willing to have their respective patients with AD participate in a therapeutic trial for aducanumab. Our findings suggest a greater need for caregiver education and involvement, especially among underrepresented racial groups (NHPI) that are less familiar with aducanumab and AD treatments. Future studies should highlight underrepresented and underserved populations specifically, as current studies of primarily Caucasian populations report aducanumab concerns inconsistent with our study’s findings. Since aducanumab familiarity may not be directly associated with an accurate level of knowledge, additional medication education is necessary by clinicians and informed healthcare professionals to deliver accurate, up-to-date information to guide treatment decision-making and understanding.
